# ^1^H-NMR metabolomic profiling reveals a distinct metabolic recovery response in shoots and roots of temporarily drought-stressed sugar beets

**DOI:** 10.1371/journal.pone.0196102

**Published:** 2018-05-08

**Authors:** Rita Wedeking, Mickaël Maucourt, Catherine Deborde, Annick Moing, Yves Gibon, Heiner E. Goldbach, Monika A. Wimmer

**Affiliations:** 1 Department of Plant Nutrition, INRES, University of Bonn, Bonn, Germany; 2 UMR1332 Biologie du Fruit et Pathologie, INRA, Université Bordeaux, Villenave d’Ornon, France; 3 Plateforme Métabolome Bordeaux- MetaboHUB, Centre de Génomique Fonctionnelle - IBVM, Villenave d’Ornon, France; Institute of Genetics and Developmental Biology Chinese Academy of Sciences, CHINA

## Abstract

Yield formation in regions with intermittent drought periods depends on the plant’s ability to recover after cessation of the stress. The present work assessed differences in metabolic recovery of leaves and roots of drought-stressed sugar beets with high temporal resolution. Plants were subjected to drought for 13 days, and rewatered for 12 days. At one to two-day intervals, plant material was harvested for untargeted ^1^H-NMR metabolomic profiling, targeted analyses of hexose-phosphates, starch, amino acids, nitrate and proteins, and physiological measurements including relative water content, osmotic potential, electrolyte leakage and malondialdehyde concentrations. Drought triggered changes in primary metabolism, especially increases in amino acids in both organs, but leaves and roots responded with different dynamics to rewatering. After a transient normalization of most metabolites within 8 days, a second accumulation of amino acids in leaves might indicate a stress imprint beneficial in upcoming drought events. Repair mechanisms seemed important during initial recovery and occurred at the expense of growth for at least 12 days. These results indicate that organ specific metabolic recovery responses might be related to distinct functions and concomitant disparate stress levels in above- and belowground organs. With respect to metabolism, recovery was not simply a reversal of the stress responses.

## Introduction

Yield stability under changing and variable water conditions is of strategic importance in securing food for a still growing world population [1]. Although the yearly amount of precipitation in Europe changed only marginally during the last 100 years, meteorologists observe a larger shift between the seasons, i.e. longer drought periods occur during spring and summer [2]. Hence, crops are under increasing strain to cope with changing environmental conditions still maintaining high productivities. In sugar beet production, climate change scenarios for the period 2021–2050 predict drought-related yield decreases of about 1 t sugar ha ^-1^ in northern France, Belgium and west/central Poland [3]. While the impact of progressive drought on the physiological and metabolic processes of plants is frequently described, studies of metabolic plant responses to rehydration are limited [4,5]. Rapid recovery after drought spells is a desirable trait for crops, particularly since plants are usually exposed to repeated drought events throughout their life cycle, which may even progress in severity.

Recovery defines the time period after cessation of a stress until a new physiological and metabolic equilibrium is established and is a crucial step in metabolism. In response to a stress, physiological adaptations and modifications of the metabolism lead to the accumulation of metabolites, including protective compounds, that may confer tolerance or resistance to drought stress [6]. Once the stress is terminated, recovery processes set in, and the plant must strike a balance between the investment of resources into damage repair, maintained acclimation (priming for upcoming stress events), or into new growth/reproduction (resetting) [7]. While resetting maximizes growth and yield under favorable conditions, it carries the risk of major and possibly fatal damage if the stress recurs. Maintained acclimation, on the other hand, makes the plant “alert” for future stress events (stress imprint), but comes at the cost of reduced growth or development and reduced yield [7]. The latter authors argue that such a “stress imprint” is a rather rare event and that return to the initial (pre-stress) metabolic and physiological state is more common, but metabolic studies confirming this hypothesis are still scarce. It seems likely that intermediate forms of recovery (to some extent, but not to the pre-stress level) might be more common, since they would represent the most promising response strategy at least in regions where recurring stresses are usually erratic and not predictable. Indeed, in a recent study it was shown that drought stress and subsequent recovery in *Medicago* had distinct dynamics and were independently regulated [5].

Under recovery the metabolic energy flows into preparation and adjustment for the reactivation of photosynthesis and respiration [8], highly-synchronized and sensitive processes that are delicate to manage. For sugar beet, available studies of recovery processes after a drought spell are mainly restricted to describe changes of the biochemical composition and sucrose accumulation of the root [9,10], or handle the effect of transient and continuous drought on yield, photosynthesis and carbon discrimination [11]. To maintain a high yield, it is of particular importance that leaves and roots recover quickly after drought to assure water and nutrient uptake and to continue sugar accumulation. A better understanding of the similarities and specificities of leaves and roots in metabolic adjustment and recovery after a transient drought is required and is a major objective of the present study.

The current work aimed at the identification and characterization of major metabolites of the primary metabolism to uncover the organ specific metabolomic strategy of transiently drought-stressed sugar beets. The integrated use of metabolomic tools such as proton nuclear magnetic resonance spectroscopy (^1^H NMR) and systems biology are powerful tools to gain a comprehensive overview of the involved pathways and the identification of crucial compounds of the metabolic response [12]. Since plant metabolites are extremely diverse in their biological function as well as in their chemical structure, ^1^H-NMR analysis is an excellent tool to study not only the composition of compounds of the plant metabolism, but also dynamic aspects as recently reviewed by [13].

## Material and methods

### Plant growth conditions

Seeds of *Beta vulgaris* L. cultivar Pauletta (KWS Saat AG, Einbeck, Germany) were cultivated in a complete randomized block with four biological replicates for each harvest day and treated as described in Wedeking et al. [14]. Plants were grown under controlled conditions at 24°C day / 18°C night temperature, 75 ± 10% relative humidity and a photoperiod of 16 h light (> 250 μmol m^2^ s^-1^: SON-T Agro, 400W, Philips, Germany). Three times a day water and nutrients (1.4‰ Hakaphos blue, Compo Expert, Münster, Germany) were supplied for 3 min each using a time controlled, automated irrigation system. During the experimental period plants were kept free of pests and diseases by integrated plant protection. To avoid uncontrolled side effects triggered by circadian rhythm and metabolite concentrations plants were sampled 2 h after beginning of the photoperiod.

### Treatments and sampling

Treatments started at BBCH 14–15 [15], i.e. when 4–5 leaves were visible. Plants were then either watered as before (control) for 25 d or subjected to intermittent drought, i.e. 13 d of progressive desiccation with subsequent rewatering for 12 d (recovery period, day 14–25). For control plants, a relative soil water content (SWC) of 65 ± 1% (w/w; based on substrate FW) was considered as optimum water supply (Wedeking et al. [14]). Under desiccation, the SWC decreased slowly to 52 ± 4% (w/w) within 7 d, then quickly to 27 ± 1% (w/w; day 9), and reached 17 ± 1% (w/w) at day 13. Under rewatering, SWC returned to 54 ± 1% (w/w) after 9 d. The SWC did not reach the initial 65%, because the amount of water used for daily watering was not adjusted for plant growth in the course of the experiment, and because plants were always harvested several hours after the last water application, when plants had already used up some of the water. To confirm that neither water logging nor water deficit occurred during the experimental period, a subset of 15 pots was weighed every second day.

Plants were harvested every other day during desiccation and rewatering and in addition daily during the first 4 d after the onset of rewatering. The youngest fully expanded leaf pair (YEL) and the root part 1.5 cm below the crown were sampled for leaf and root analysis, respectively (S1 Fig). The first leaf of the YEL pair was used for the metabolite analysis and the determination of malondialdehyde (MDA). The second leaf was halved. One half was used for the analysis of osmotic potential (OP) and from the other half, six leaf discs (diameter 9 mm) were punched out avoiding leaf veins and used for the determination of relative water content (RWC) and electrolyte leakage (EL). For the analysis, plant material was either directly processed (RWC, EL), stored at -20°C (OP) or immediately frozen and ground under liquid nitrogen (leaf, MDA, hexose-phosphates, starch) or lyophilized (root), and stored at -80°C until further analysis. For ^1^H-NMR analysis leaf material was also lyophilized and stored at -80°C until the analysis.

### Analysis of biomass, relative water content, electrolyte leakage and osmotic potential

Fresh weights (FW) of leaves and roots were recorded directly after harvest and dry weights (DW) after drying at 70°C until constant weight was reached. The determination of EL, RWC, and OP was determined as previously described [14].

### Malondialdehyde determination

The analysis of MDA, a marker of lipid peroxidation, was based on the thiobarbituric acid assay according to Hodges et al. [16] with modifications. All solutions were prepared fresh before use and samples were determined in duplicate. For the extraction, 20 mg of frozen ground plant material were homogenized with 500 μL 0.1% trichloroacetic acid (TCA). After centrifugation (*3*,*500 g*, 15 min, room temperature) 150 μL of the supernatant was mixed either with 150 μL of reagent 1 (Reagent 1 (R1): 0.01% 2,6-di-tert-butyl-4-methylphenol in 20% (w/v) TCA), or reagent 2 (Reagent 2 (R2): R1 plus 0.65% 2-thiobarbituric acid). All samples were heated (95°C; 30 min), cooled, and briefly centrifuged (<1 min, *3*,*500 g*, room temperature) and immediately measured at 440, 532 and 600 nm using a microplate reader (Power Wave XS2, Bad Friedrichshall, Germany). Malondialdehyde equivalents in nmol mL^-1^ were calculated according as follows

A=[(Abs532R2−Abs600R2)−(Abs532R1−Abs600R1)] (1)

B=[(Abs440R2−Abs600R2)×0.0571] (2)

MDA[nmolmL−1]=[(A−B)]41448×10^6 (3)

where 0.0571 corresponds to the ratio of the molar absorbance of 1^−10^ mM sucrose at 532 nm and 440 nm and, 41448 refers to the molar extinction coefficient (ε) of MDA calculated for d_100μL_ = 0.264.

### Proton NMR metabolomic profiling

Polar metabolites were extracted from leaf and root samples. Briefly, polar metabolites were extracted from 20 mg of ground lyophilised powder with an ethanol-water series at 80°C (adapted from Moing et al. [17]) using a pipetting robot (Hamilton, Bonaduz, Switzerland) with two technological replicates per sample. The supernatants were combined, dried under vacuum and lyophilised. Each lyophilised extract was solubilized in 500 μL of 100 mM deuterated potassium phosphate (KOD) buffer solution pH 6.0, containing 3 mM ethylene diamine tetraacetic acid disodium salt (EDTA), adjusted with KOD solution to pH 6 when necessary, and lyophilised again. The lyophilised titrated extracts were stored in darkness under vacuum at room temperature, before ^1^H-NMR analysis was completed within one week.

For ^1^H-NMR analysis, 500 μL of D_2_O with sodium trimethylsilyl [2,2,3,3-d4] propionate (TSP, 0.01% mg/mL final concentration for chemical shift calibration) were added to each lyophilised titrated extract. The mixture was centrifuged at *17*,*700 g* for 5 min at room temperature. The supernatant was then transferred into a 5 mm NMR tube for acquisition. Quantitative ^1^H-NMR spectra were recorded at 500.162 MHz and 300 K on a Avance III spectrometer (Bruker Biospin, Wissembourg, France) using a 5-mm ATMA broadband inverse probe, a 90° pulse angle and an electronic reference for quantification (Digital ERETIC, Bruker TopSpin 3.0). The assignments of metabolites in the NMR spectra were made by comparing the proton chemical shifts with literature [18] or database values (MERy-B: [19,20]; HMDB: [21]; BMRB http://www.bmrb.wisc.edu), by comparison with spectra of authentic compounds and by spiking the samples. For assignment purposes, ^1^H-^1^H COSY, ^1^H-^13^C HSQC and ^13^C NMR spectra were acquired for selected samples. The identified metabolites are indicated in Tables 1 and 2, with identification levels according to MSI [22]. For absolute quantification three calibration curves (glucose and fructose: 1.25 to 50 mM, glutamate and glutamine: 0 to 15 mM) were prepared and analysed under the same conditions. The glucose calibration was used for the quantification of all compounds, as a function of the number of protons of selected resonances except fructose, glutamate and glutamine that were quantified using their own calibration curve. The metabolite concentrations were calculated using AMIX (version 3.9.10, Bruker) and Excel (Microsoft, Redmond, WA, USA) software.

**Table 1:** Chemical shifts used for identification and quantification of integrated soluble sugars and organic acids in ^1^H-NMR spectra of beet root and leaf polar extracts (in D_2_O, pH_apparent_ 6.0), expressed as relative values to the TSP resonance at 0 ppm. s: singlet, d: doublet, dd: doublet of doublets, t: triplet, m: multiplet.

Compound	Group	Multiplicity	δ^1^H (ppm) D_2_O pH6	Root (R) Leaf (L)	Identification status^a^	Integrated range (ppm) used for quantification
*Integrated soluble sugars*						
Fructose	α(C3H+C5H)+βC5H	m	4.12	L	2	4.115 +/- 0.011
α-Glucose	C1H	d	5.22	L R	1	5.234 +/- 0.014 5.225 +/- 0.009
β-Glucose	C1H	d	4.65	L R	1	4.651 +/- 0.020 4.648 +/- 0.015
β-Glucose	C2H	t	3.25	LR	1	
Raffinose	Galactosyl-C1H	d	5.00	L R	1	5.002 +/- 0.011 5.003 +/- 0.010
Sucrose	Glucopyranosyl-C1H	d	5.42	LR	1	5.424 +/- 0.024
UDP-glucose-like	C1H ribose	m	5.98	R	3	5.984 +/- 0.012
*Organic acids*						
Citric acid	C2H_2_ + C4H_2_	dd	2.58	L R	1	2.577 +/- 0.026 2.596 +/- 0.017
Formic acid	C1H	s	8.46	L	2	8.459 +/- 0.004
Fumaric acid	C2H + C3H	s	6.52	R	2	6.523 +/- 0.007
Malic acid	C2H	dd	4.30	L R	1	4.307 +/- 0.021 4.302 +/- 0.020

^a^ Identification level: 1, Identified compounds (checked with standard); 2, Putatively annotated compounds; 3, Putatively characterized compound classes; 4, Unknown.

**Table 2:** Chemical shifts used for identification and quantification of amino acids, other amino compounds and other compounds in ^1^H-NMR spectra of beet root and leaf polar extracts (in D_2_O, pH_apparent_ 6.0), expressed as relative values to the TSP resonance at 0 ppm. s: singlet, d: doublet, dd: doublet of doublets, t: triplet, m: multiplet.

Compound	Group	Multiplicity	δ^1^H (ppm) D_2_O pH6	Root (R) Leaf (L)	Identification status^a^	Integrated range (ppm) used for quantification
*Amino acids and other amino compounds*						
Alanine	C3H_3_	d	1.48	L R	1	1.484 +/- 0.015 1.484 +/- 0.015
Arginine	C7H_2_	m	1.63	L	1	1.635 +/- 0.027
Asparagine	½ (C3H_2_)	m	2.88	L R	1	2.979 +/- 0.006 2.882 +/- 0.016
Aspartic acid	½ (C3H_2_)	½ dd	2.82	L R	1	2.819 +/- 0.027 2.800 +/- 0.012
GABA	C2H_2_	t	2.30	R	1	2.303 +/- 0.003
Glutamic acid	C3H_2_	m	2.36	L R	1	2.360 +/- 0.007 2.359 +/- 0.007
Glutamine	C4H_2_	m	2.46	L R	1	2.459 +/- 0.025 2.458 +/- 0.025
Isoleucine	C6H_3_	s	1.02	L R	1	1.022 +/- 0.006 1.020 +/- 0.006
Phenylalanine	C5H + C6H	m	7.41	L R	1	7.405 +/- 0.050 7.409 +/- 0.050
Proline	C4H_2_	m	2.33	L R	2	2.334 +/- 0.004 2.333 +/- 0.005
Pyroglutamic acid	C2H	dd	4.17	L R	1	4.178 +/- 0.018 4.172 +/- 0.014
Serine	C2H_2_	m	3.97	L R	1	3.967 +/- 0.003 3.981 +/- 0.008
Tryptophan	C7H	d	7.55	L R	1	7.541 +/- 0.016 7.546 +/- 0.015
Tyrosine	C3H_2_	d	6.91	L R	1	6.905 +/- 0.020
	C2H_2_	d	7.19	L R	1	7.189 +/- 0.008
Valine	C4H_3_	d	1.04	L R	1	1.045 +/- 0.013 1.046 +/- 0.014
	C5H_3_	d	1.00	LR	1	
Choline	N-C3H_3_+N-C4H_3_+N-C5H_3_	s	3.20	R	1	3.205 +/- 0.006
Glycine betaine	N-C3H_3_+N-C4H_3_+N-C5H_3_	s	3.27	L R	1	3.269 +/- 0.010 3.267 +/- 0.015
	C2H_2_	s	3.83	LR	1	
Trigonelline	C2H	s	9.17	L	2	9.127 +/- 0.008
	N-CH_3_	s	4.44	L	2	
*Other compounds*						
Xanthine_like	C2H	s	8.46	R	3	8.459 +/- 0.004
UnknownS8.29		s	8.29	L	4	8.297 +/- 0.018
UnknownS5.35		s	5.35	R	4	5.395 +/- 0.004
UnknownS5.25		s	5.25	L	4	5.254 +/- 0.006
UnknownS2.75		s	2.75	R	4	2.755 +/- 0.006
UnknownD1.84		d	1.84	L	4	1.842 +/- 0.009

^a^ Identification level: 1, Identified compounds (checked with standard); 2, Putatively annotated compounds; 3, Putatively characterized compound classes; 4, Unknown.

### Ethanolic extraction of metabolites

For the ethanolic extraction 20 mg and 10 mg of leaf and root were used. The powdered material was extracted as previously described in Wedeking et al. [14].

### Hexose-phosphates determination

Hexose-phosphates, glucose-6-phosphate (G6P) and fructose-6-phosphate (F6P), were determined based on Gibon et al. [23] with modifications. For the analysis of G6P, 75 μL of assay mix 1 consisting of 0.2 M tricine/KOH pH9 with 10 mM MgCl_2_, 100 u mL^-1^ glucose-6-phosphate dehydrogenase grade II (G6PDH, E.C. 1.1.1.49), 2.5 mM NADP and ultrapure water were added to 5 μL of the ethanolic extract. After 20 min incubation at room temperature, 20 μL of 0.5 M sodium hydroxide (NaOH) was added and samples were incubated for 10 min at 98.5 °C in a dry bath. Then, 20 μL of 0.1 M Tricine/KOH pH 9 containing 0.5 M hydrogen chloride (HCl) were added to the cooled and briefly centrifuged (1 min, *1*,*000 g*, RT) samples. Finally, assay mix 2 consisting of 0.2 M Tricine/KOH pH 9 with 10 mM MgCl_2_, 500 u mL^-1^ G6PDH grade I (EC: 1.1.1.49), 200 mM EDTA pH 8, 250 mM G6P, 10 mM phenazine methosulfate (PMS) and 10 mM thiazolyl blue tetrazolium bromide (MTT) were added, and samples were immediately measured at 570 nm (37 °C, 30 s) until rates were stabilized using a microplate reader (Safas M96, Monaco). For the analysis of F6P, 30 μL of assay mix 1 consisting of 0.2 M Tricine/KOH pH 9 with 10 mM MgCl_2_, 100 u mL^-1^ G6PDH grade II, 2.5 mM NADP and ultrapure water were added to 10 μL of the ethanolic extract and incubated for 20 min at room temperature. Subsequently, 10 μL 0.25 M HCl were added and samples were incubated for another 5 min at room temperature, before 10 μL of assay mix 2, consisting of 100 u mL^-1^ G6PDH grade II, 20 u mL^-1^ phosphoglucoisomerase (PGI, EC: 5.3.1.9) and ultrapure water were added. After incubation for 20 min at RT, 20 μL 0.5 M NaOH were added and samples were incubated for 10 min at 98.5 °C in a dry bath. Before 20 μL of 0.1 M Tricine/KOH pH 9 with 0.5 M HCl were added, samples were cooled and briefly centrifuged (1 min, *1*,*000 g*, RT). Finally, 52 μL of assay mix 3 consisting of 0.2 M Tricine/KOH pH 9 with, 10 mM MgCl_2_, 1000 u mL^-1^ G6PDH grade I, 200 mM EDTA pH 8, 250 mM G6P, 10 mM PMS and 10 mM MTT were added and samples were immediately measured at 570 nm (37 °C, 30 s) until rates was stabilized using a microplate reader (M96, Safas; Monaco). Both, G6P and F6P concentrations were calculated based on the regression equations of standard solutions (0 to 10 μM G6P and F6P respectively).

### Starch determination

Starch was determined in form of glucose equivalents according to Hendriks et al. [24] using the pellet form the ethanolic extraction. After resuspension in 400 μL 0.1 M NaOH, samples were heated at 95°C for 30 min, cooled, homogenized and centrifuged (*1*,*000 g*, 5 min). Subsequently, samples were hydrolyzed by adding 0.5 M HCl with acetate/0.1 M NaOH buffer, pH 4.9. For starch degradation, 35 μL of the thoroughly mixed sample were transferred into a new 96-well plate, adding 65 μL of a degradation mix consisting of 250 μL amyloglucosidase (EC: 3.2.1.3), 3 μL α-amylase (EC: 3.2.1.1) and 50 mM acetate buffer pH 4.9. Finally, samples were digested for 16 h at 37°C. Before the determination of the glucose as previously in Wedeking et al. [14], the plate was centrifuged (*1*,*000 g*, 10 min, room temperature).

### Nitrate determination

For the analysis of nitrate (NO_3_-) root and leaf samples were diluted with 0.1 M potassium phosphate (KOH) buffer, pH 7.5. Standards (SDs) were prepared with 10 μL (0–2 mM mL^-1^ sodium nitrate in 96% EtOH). For the analysis, 95 μL of the assay mix containing nitrate reductase (NR; EC: 1.7.1.2) were added to the samples. Blanks were prepared with the assay mix *without* NR to determine the nitrite amount in the samples. In case of the assay mix for the blanks, NR was replaced with 0.1 M KOH, pH 7.5. Afterwards, all samples were homogenized and incubated for 30 min at RT in the dark. Then, 15 μL of 0.25 mM PMS were added, samples were mixed again and incubated for another 20 min at room temperature. Subsequently, 60 μL of 1% sulfanilamide (w/v) in 3 M phosphoric acid and 60 μL of 0.02% (w/v) N(1-Naphtyl)ethylemdiamine dihydrochloride (NNEDA) in 3 M phosphoric acid were pipetted and samples were mixed. After 10 min of incubation in the dark, samples were measured immediately at 540 nm (M96, Safas, Monaco).

### Total amino acids and total protein determination

Total amino acids (AA_t_) were analysed as described in Cross et al. [25] with modifications. For the analysis, 3 μL of ethanolic extract for all samples and SDs (0–1 mM mL^-1^ glutamate sodium salt in 70% EtOH (v/v) 0.1 M HEPES/KOH, pH 7) were added with 15 μL 0.1 M sodium borate buffer, pH 8, 100 μL of ultrapure water and finally 90 μL 0.1% fluorescamine (w/v) in acetonitrile. Due to its light sensitivity fluorescamine was added in the last pipetting step, and the fluorescence was measured after incubation for 5 min at room temperature in the dark, at 405 nm for the excitation and at 485 nm for the emission (Xenius, Safas, Monaco). The glutamate SD was always prepared fresh. Total soluble protein was determined according to Bradford et al. [26].

### Data analysis

Statistical analyses were performed with SPSS 23.0 (SPSS Inc., New York, USA). Significant differences between the treatments were analyzed using a non-parametric test for independent scores. Hence, a one-factorial ANOVA according to Kruskal-Wallis (Duncan, α = 0.05) with the stepwise step-down procedure, was performed. To explore the multidimensional data set, a correlation based principal component analysis (PCA) was performed. A Kaiser-Meyer-Olkin (KMO) value of >0.80 and a significant Bartlett test (p<0.001) for sphericity indicated that PCA after unit variance scaling was suitable. The analysis was done with a matrix of 84 samples for each plant part (2 treatments, 14 harvests, 3 biological replicates), 2 factors (treatment, day) and 27 or 26 variables for leaf and root, respectively. Rotated orthogonal components (Varimax rotation) with eigenvalues >1 were extracted and relative scores were determined. Values calculated for the heatmaps represent the change of each analysed metabolite relative to the control for the respective day.

## Results

### Plants overcome drought-induced impairments of plant water status and membrane stability

**Fig 1:**
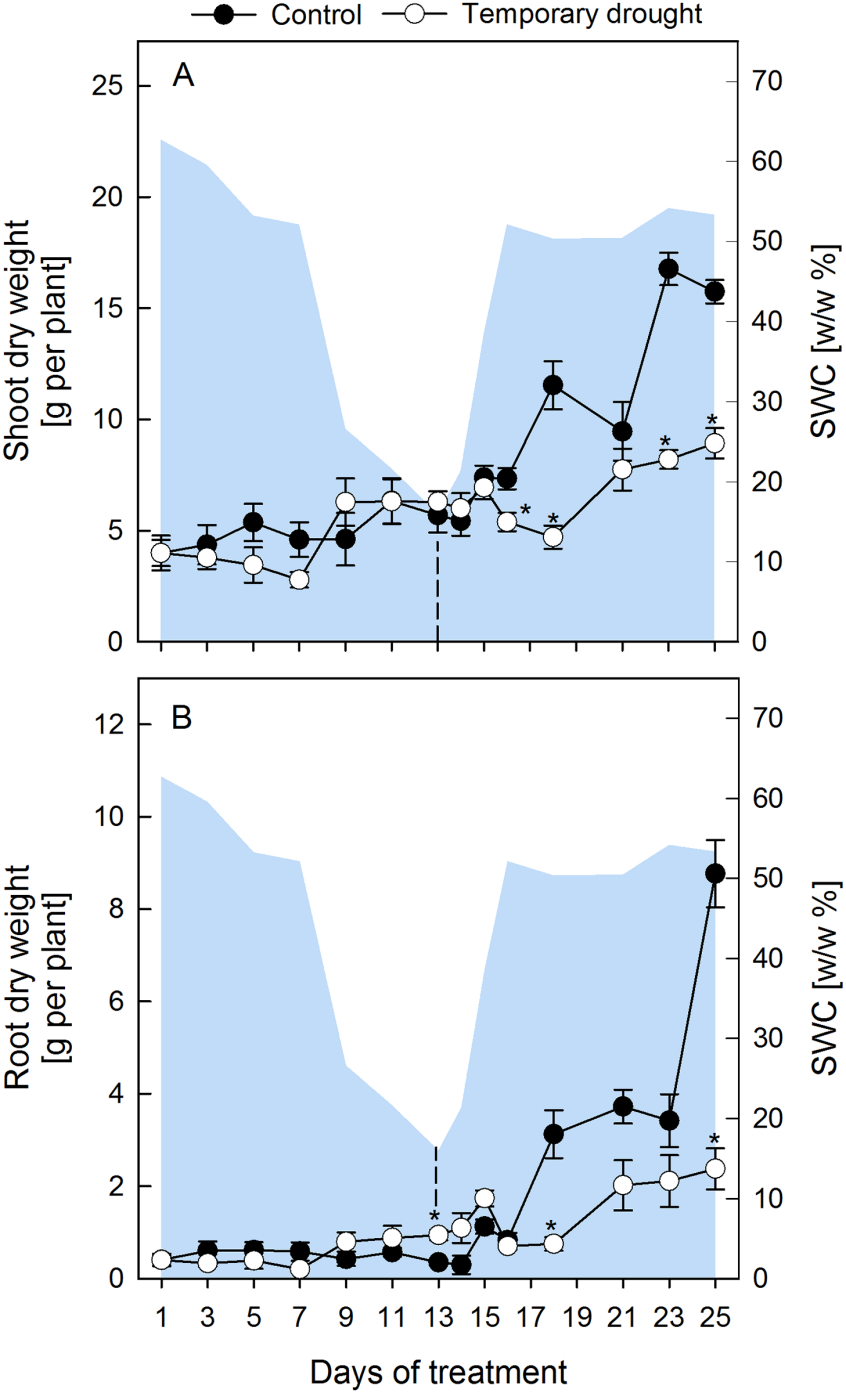
Change of biomass of sugar beet shoots and roots. Plant dry weight of sugar beets shoots (A) and roots (B) of control (closed circles) and rewatered (open circles) plants. Plants were rewatered after 13 d as indicated by the dashed line. The area plot represents the gravimetric relative soil water content (SWC w/w %). All values are means ± s.e. (n = 4). Significant differences to the control plants (Duncan, α = 0.5) are indicated by *P < 0.05.

**Fig 2:**
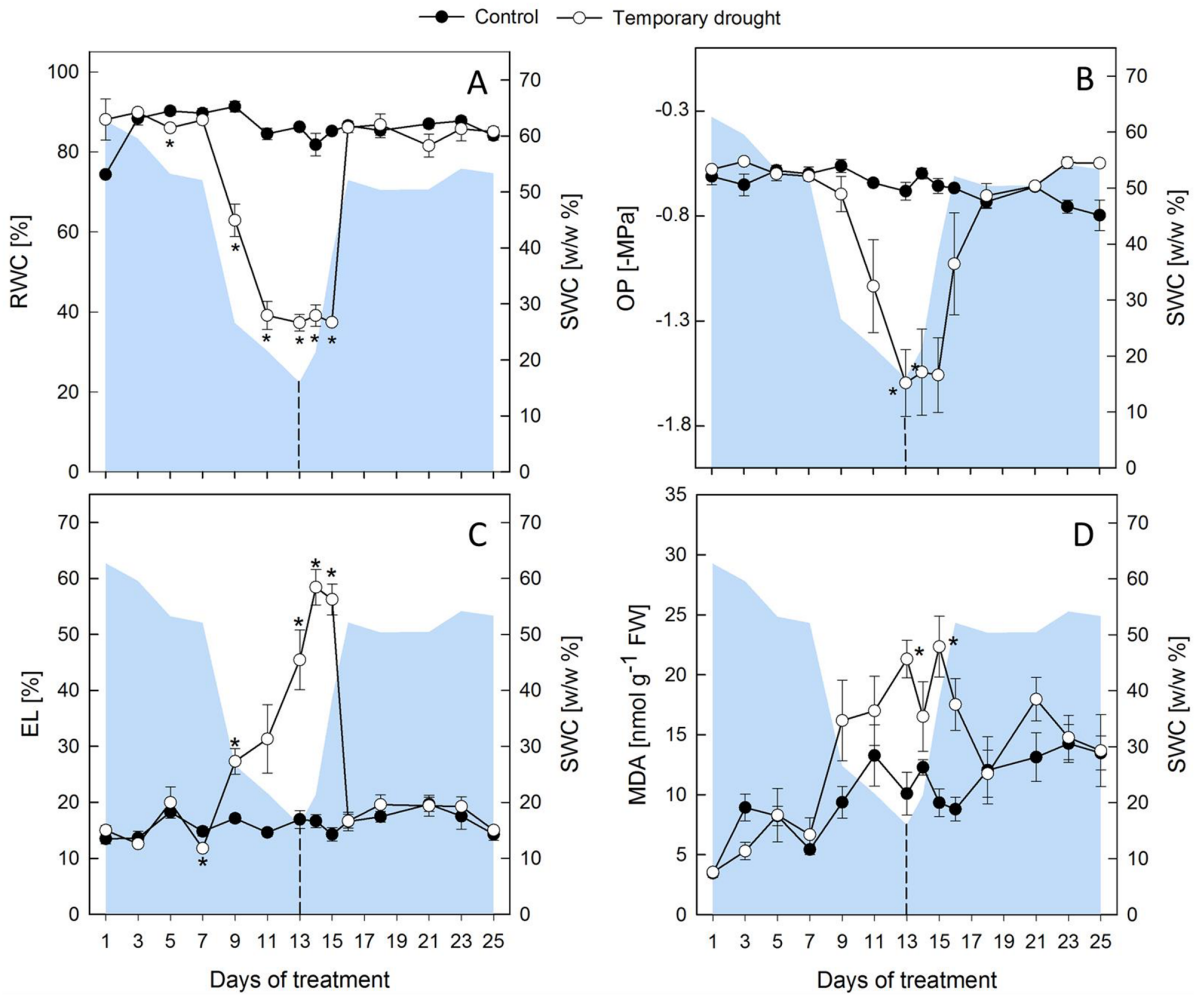
Plant water status and indicators of membrane damage of sugar beet leaves. Relative water content (RWC, A) osmotic potential (OP, B), electrolyte leakage (EL, C) and malondialdehyde (MDA) concentrations (D) of leaves under regular water supply (closed circles) and temporary drought (open circles). The area plot represents the gravimetric relative soil water content (SWC w/w %). Plants were rewatered after 13 d as indicated by the dashed line. All values are means ± s.e. (n = 4). Significant differences to the control plants (Duncan, α = 0.5) are indicated by *P < 0.05.

During progressive drought, the relative soil water content (SWC) decreased slowly within the first 7 d and then faster until d 13 (Fig 1). Under these conditions shoot dry weight (DW) was not significantly reduced compared to controls (Fig 1), which developed slowly from BBCH 16–17 (6–7 leaves, d 1) to BBCH 17–19 (7–9 leaves, d 25), but plants subjected to drought had a significantly higher root DW at the end the drought period (Fig 1). The RWC of leaves (Fig 2) dropped significantly after d 7 and reached a minimum value of 37 ± 2% on d 11 of drought, while the largest decrease in OP (Fig 2B) was observed between d 9 and 13 of drought with final values of -1.56 ± 0.2 MPa. Leakage (Fig 2) and MDA (Fig 2), both indicators of membrane damage due to lipid peroxidation, were measured and first signs of membrane damage in the shoot were observed after 7–9 d of desiccation.

After the onset of rewatering, younger leaves of stressed plants regained turgor within 2 d, but oldest leaves did not fully recover until the end of the experiment (S2 Fig). A lag period of 5 d was observed before stressed plants started regrowth, but they maintained a low relative growth rate of only 26% (shoots) and 31% (roots) of the control growth rates between d 14 and 25 (Fig 1). Both RWC and OP showed a lag-phase of 2 d after the onset of rewatering, before they started to recover, and then reached control levels within 1 d (RWC) and 2 d (OP), respectively (Fig 2). MDA returned to control levels within 4–6 d. However, EL continued to increase for 1–2 d into the rewatering period, but then recovered more quickly compared to MDA and reached control levels within 3 d (Fig 2).

### Temporary drought leads to changes in primary metabolism

**Fig 3:**
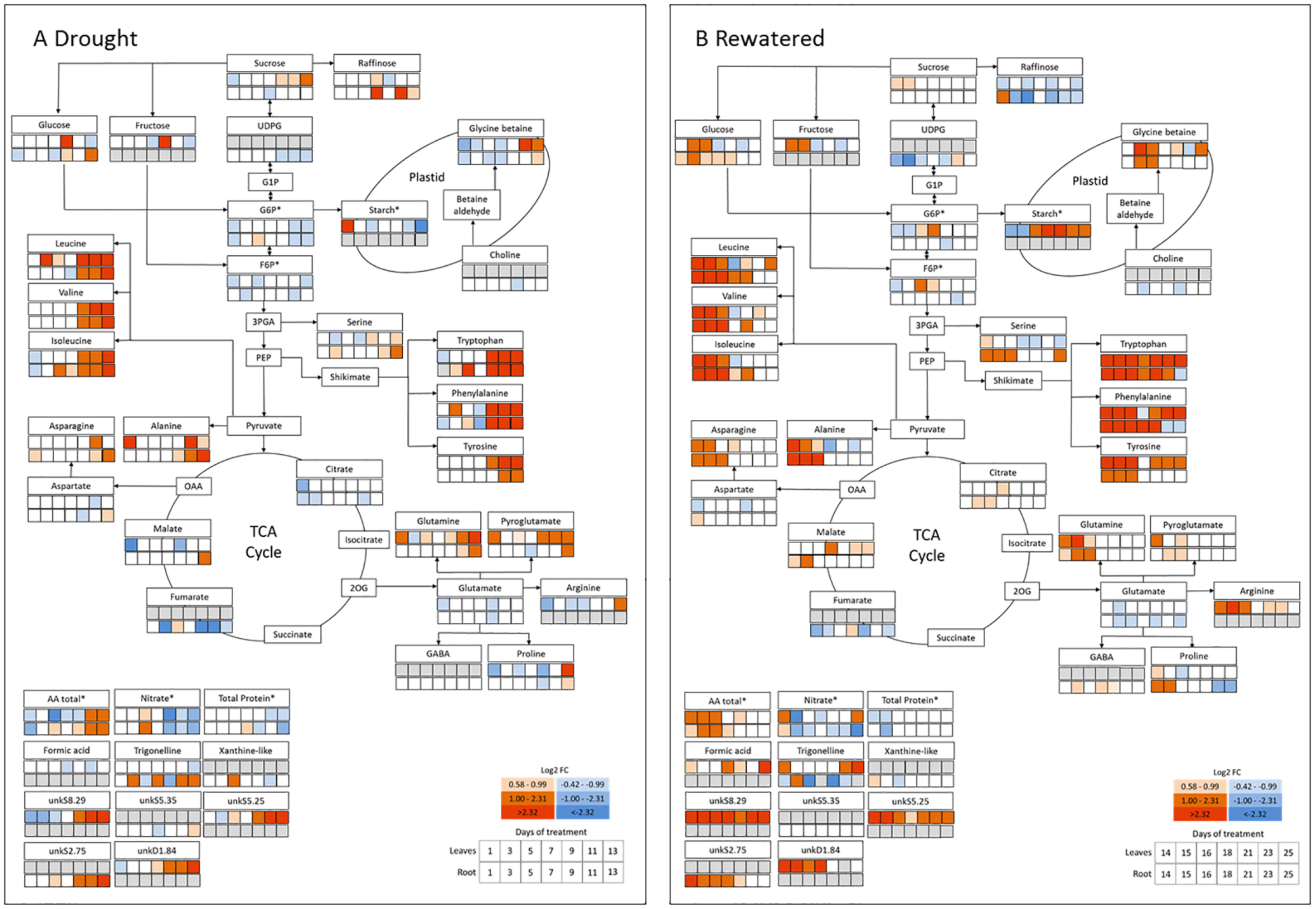
Metabolic map of the differences in the ^1^H-NMR metabolomic profile during 13 days of progressive drought with subsequent rewatering. Changes under drought (1–13 d, A) and rewatering (14–24 d, B). The first row indicates the metabolic alterations in the leaves, the second row indicate the changes in the root. Red and blue colors indicate the log_2_-transformated abundance (log2 Fold change, log_2_FC) of each metabolite relative to the controls. Grey boxes indicate that the respective metabolite was not identified (leaf only: Fructose, starch, formic acid, unkS8.29, unkS5.25, unkD1.84. Root only: UDP-glucose-like sugar, choline, GABA, xanthine-like, unkS2.75, unkS5.35.). Asterisks (*) indicate that metabolites were determined by robotized enzyme assays (G6P, F6P, starch, total amino acids (AA_t_), nitrate, total protein).

Overall, 29 metabolites were identified by ^1^H-NMR, including six carbohydrates, 15 amino acids (AA), four organic acids, two quaternary ammonium compounds one purine compound and one alkaloid (Tables 1 and 2, S3 Fig). For the comparison of drought induced changes (1–13 d) and the differences between leaves and roots (13–25 d) under rewatering, metabolic maps were created showing the log_2_-fold change between well-watered and drought-stressed/rewatered plants for each harvest day (Fig 3).

Temporary drought caused a change of several metabolites including sugars, organic acids, compatible solutes and especially AA in both organs (Fig 3). In leaves, opposite effects were observed for sucrose and starch, where sucrose levels increased, while starch concentrations decreased towards the end of the desiccation period (Fig 3, S4 Fig). The quantifiable intermediates of the TCA cycle (citrate, malate, fumarate) were only marginally affected during drought, with the exception of a reduction of fumarate levels in roots (Fig 3). Drought induced metabolic reprogramming resulted in an increase of AA_t_, as well as decreases in nitrate and protein (Fig 3). The most significant increase was observed for branched chain amino acids (BCAA: leucine, isoleucine, valine), alanine derived from pyruvate, and aromatic amino acids (AAA: tryptophan, phenylalanine, tyrosine) derived from phospho*enol*pyruvate, with the maximum change observed for phenylalanine (>200 fold in leaves, >70 fold in roots). Especially in leaves, drought-induced increases in glutamine, pyroglutamate, arginine, and proline were associated with a decrease of their precursor glutamate (Fig 3). Correspondingly, an increase of asparagine in leaves was accompanied by a decrease of its precursor aspartate. The quaternary ammonium compound glycine betaine (GB) and proline also accumulated towards the end of the stress in shoots, but only marginally in roots, where the GB increase was nevertheless associated with a decline of its precursor choline (Fig 3). Overall, the metabolic pathway map indicates that under drought stress, glycolysis and TCA cycle were rather downregulated, while levels of AA were significantly enhanced.

### Different dynamics in leaves and roots during the recovery process

The majority of the increased metabolites under drought approached or returned to control level between d 15–18 (2–5 d after rewatering, DAR), with the notable exception of starch, which increased throughout the recovery period and reached significantly higher values at the end of the experiment compared to controls (S4 Fig). Some distinct differences were observed between leaves and roots (Fig 3). In leaves, glucose (and similarly fructose) quickly dropped below controls at the beginning of rewatering, but then showed a second transient peak, from d 15–16, while in roots, glucose only slowly returned to control levels (d 23).

Citrate and malate increased slightly during rewatering in leaves and roots, while fumarate (only detectable in roots) remained lower than control levels throughout the recovery. In leaves, rewatering mostly reversed the drought induced increases of AA within 5 d of rewatering (d 18), while accumulated AA decreased more slowly, but constantly, in roots and reached control levels at d 23. Notably the AAAs, and less pronounced leucine, valine and GB, showed a second strong increase towards the final days of the rewatering in leaves, but not in roots.

A major difference between roots and leaves was the response of serine, which was more strongly induced by drought in roots, where it only slowly returned to control levels during rewatering, and also showed a strong second increase towards the end of the rewatering period, similar to the dynamics observed for AAAs in leaves. Summarized, the observed dynamics in the metabolite abundance indicate distinct alterations in the metabolic activity of the involved pathways under drought and recovery, and between leaves and roots.

**Fig 4:**
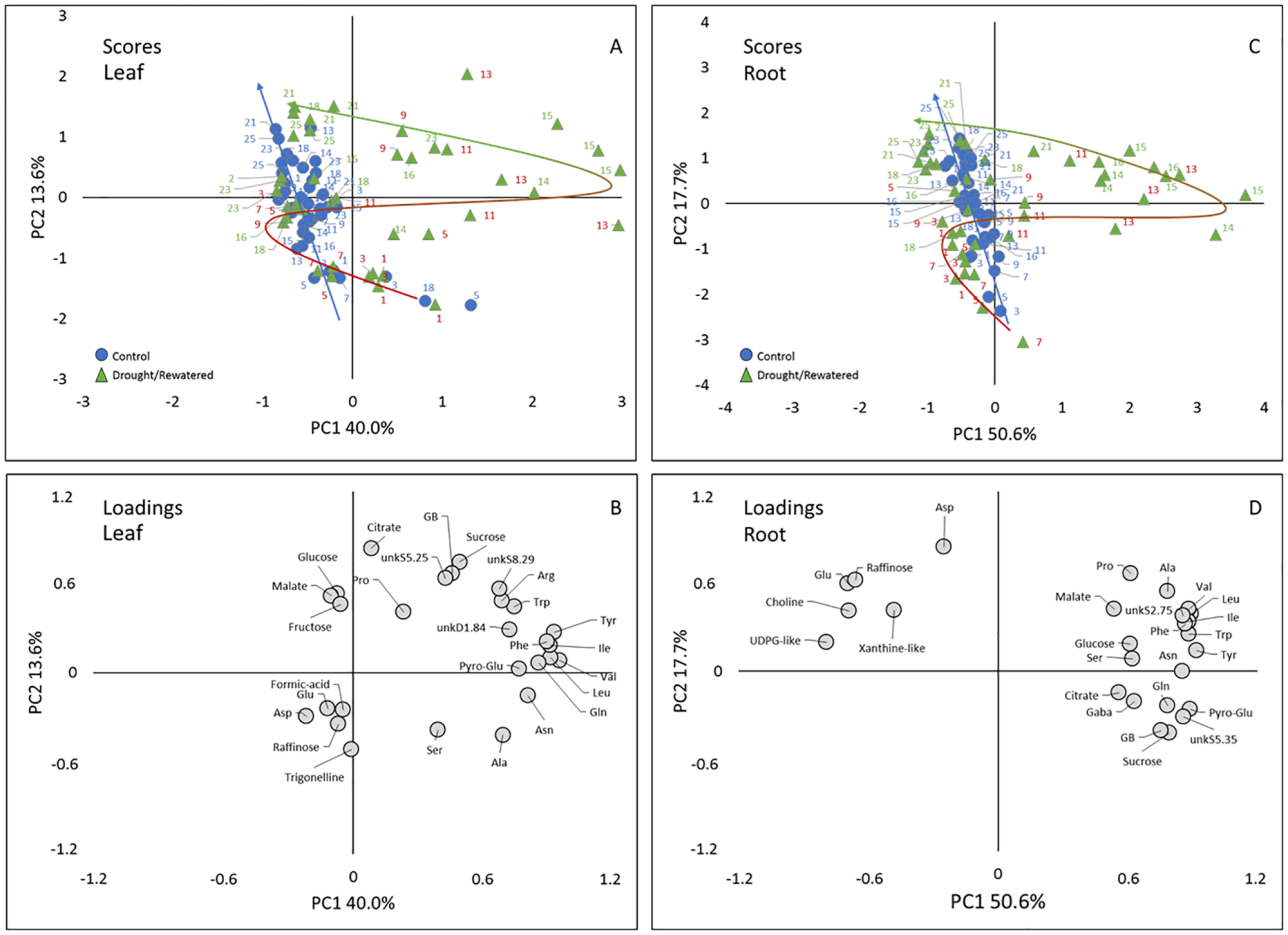
Result of the principal component analysis (PCA) of the ^1^H-NMR profiles. Scores plot of leaf (A) and root (C), loadings plot of leaf (B) and root (D); samples size n = 3. The PC1 x PC2 plots represent 53.6% and 68.3% of total variance for leaves and roots, respectively. In the scores plot circles represent control plants and triangles represents temporary stressed plants. Trajectories in the scores plot represent the temporal development of metabolic response during the treatments (blue arrow, control; bicolored arrow: red: drought, green: rewatered. Abbreviations: Asn, asparagine; Asp, asparagine; Gaba, γ-aminobutyric acid; GB, glycine betaine; Gln, glutamine; Glu, glutamate; Ile, isoleucine; Leu, leucine; Val, valine; Phe, phenylalanine; Pro, proline; Pyro-Glu, pyroglutamate; Ser, serine; Trp, tryptophan; Tyr, tyrosine; UDPG-like, uridine diphosphate glucose-like; unk, unknown compound.

In order to search for metabolites that were the most important indicators for stress and recovery, and to assess whether and how the drought induced changes were reversed under rewatering, a principal component analysis (PCA) was performed for each plant part using a matrix containing the data of 27 (leaves) and 26 (roots) quantified metabolites in 84 samples for each leaves and roots (Fig 4). The PCA allowed to visualize the separation of the different time points, and to identify the metabolites involved in the dynamic response.

For the leaf PCA (Fig 4), the first two principal components (PCs) explained 53.6% of total variability, with 40.0% for the first principal component (PC1) and 13.6% for the second principal component (PC2). In the scores plot (Fig 4), PC1 separated a large group containing control, mildly stressed (<d 9) and late rewatered (>d 15) samples on the negative side from a group containing samples taken at later stages of drought and early rewatering (d 9-15, positive side), indicating that PC1 seems related to drought stress intensity. PC2 tended to separate younger (~d 1–15, negative side), from older plants (~d 16–25, positive side), suggesting that PC2 seems related to leaf development. However, this separation was less clear than for PC1, in line with a more gradual change in metabolism throughout development. The trajectories (Fig 4) visualize the differences in metabolic patterns over time between control and drought stressed/rewatered plants.

Comparison of the scores plot and loadings plot (Fig 4) showed that the samples taken at later stages of drought and early rewatering tended to have higher contents in a range of AA including tyrosine, tryptophan, phenylalanine, pyroglutamate, leucine, isoleucine, valine and glutamine. Along PC2, the younger plants seemed characterized by higher contents of trigonelline, and the older ones by higher contents of citrate.

For the root PCA (Fig 4), the first two PCs explained 68.3% of total variability (PC1 50.6%, PC2 17.7%). In the scores plot (Fig 4), PC1 separated one large group containing control plants, mildly stressed (<d 11) and plants of late rewatering (>d 16) on the negative side, from a smaller group characterized by samples taken at later stages of progressive drought and early rewatering (d 11–16) on the positive side, which indicates that PC1 also seems related to stress intensity. PC2 tended to progressively separate samples at early stages on its negative side from samples at late stages of development on its positive side. Comparison of the scores plot (Fig 4) and loadings plot (Fig 4) showed that the samples taken at later stages of drought and early rewatering tended to have higher contents in a range of amino acids similarly to leaves and also sucrose and GB. Roots at the later stages had higher contents in aspartate, glutamate and raffinose.

The PCA of both roots and leaves confirms that major stress-induced metabolic changes occurred during the final 3–5 d of progressive drought and lasted for another 3 d into the recovery period, which exactly mirrors the response of water relations (RWC, OP) and of membrane damage (EL, MDA) during the stress and the recovery phase. In both organs the rewatered samples showed a reversed trajectory (Fig 4) and rewatered samples clustered again with controls between d 16–17, indicating the transitory nature of the metabolic changes triggered by progressive drought. However, in roots, samples taken between d 23–25 showed a tendency to separate again from the control plants along PC1 (Fig 4), suggesting that after 10 DAR (d 23), roots were metabolically distinct from non-stressed conditions, whereas this did not seem to be the case in leaves.

## Discussion

### Damage repair was important during recovery and involved glycine betaine

Drought stress triggers various physiological and biochemical responses in plants. For sugar beets, which have only a limited ability to regulate transpiration [27], the adaptation of the metabolism after a drought spell is especially relevant. While drought induced metabolic changes have been extensively described [28–30], little is known about the dynamics and completeness of metabolic recovery in *Beta vulgaris*. In the present study, young plants suffered from significant membrane damage and lipid peroxidation after 13 d of progressive drought before the onset of rewatering. Based on a previous study, sugar beets are severely, but not lethally stressed under these conditions [14].

The typical accumulation of primary metabolites such as soluble carbohydrates, organic and amino acids as well as amides was observed at the end of the drought period in both organs (Fig 3) and is consistent with other studies [4]. While after 12 d of rewatering physiological parameters had returned to control levels, metabolites, especially AAAs, recovered only slowly. This is not surprising, since many processes need to be rearranged under rewatering to reach a new balance, and metabolic adjustments are needed to coordinate investment of resources into damage repair and resumed growth [31]. In addition, leaves ensure photosynthesis to maintain energy supply, while roots warrant water and nutrient uptake. This requires different sets of metabolites even under well-watered conditions, and during and after drought, resources need to be re-distributed to ensure efficient recovery strategies in each organ [32]. The distinct functions of leaves and roots likely result in different stress levels and consequently distinct dynamics of metabolic responses as outlined below.

Lipid peroxidation caused by reactive oxygen species (ROS) is commonly observed under severe drought. As functional membranes are indispensable for photosynthesis, nutrient and water uptake or respiration, it can be assumed that the repair of membranes has priority during recovery. However, detoxification of ROS is an energy consuming process and requires large amounts of reductive power for enzymatic and non-enzymatic scavenging [33]. The continued increase of EL in leaves during the first two days of rewatering and the slow recovery of MDA indicate that either the supply of reductants was not available or that ROS scavenging systems were not fully recovered shortly after the onset of rewatering.

Chen and Murata [34] argue that the fate of cellular components under stress depends on the balance between damage and repair rather than the severity of the damage alone, and that elevated ROS levels hinder repair processes, even before the damage is measurable. They suggest that compatible solutes such as GB and proline protect the protein-synthesizing machinery from oxidative stress, thus maintaining conditions under which repair mechanisms occur at high rates. Compatible solutes thus fulfil a double role by conferring osmotic adjustment (OA) under drought, as well as contributing to a high rate of repair during recovery. In *Beta vulgaris*, GB is a constitutive cytoplasmic compatible solute, and can accumulate to considerable amounts under stress conditions [33]. It is likely that leaf membranes suffer more severe damage than those of roots, since a drought-induced imbalance between photosynthetic activity and growth results in an enhanced production of ROS. In the present study, both GB and proline increased in both organs very late during the drought period, and their accumulation was higher in leaves compared to roots. In summary this may indicate that they were involved in protection rather than OA during the final phase of drought, and enabled necessary repair mechanisms as suggested by Chen and Murata [35].

Elevated GB levels in sugar beet roots could negatively affect sugar yield in two different ways. Firstly, GB reacts with sucrose during processing and thus impairs sugar crystallization [36] and secondly, its biosynthesis is energy consuming, since the synthesis of 1 mol GB requires approximately the same energy input as 1 mol sucrose [37]. Hence, energy and photosynthates used in this reaction are neither available for sucrose storage nor for other processes related to economic yield. In the present study GB concentrations returned to control levels within 12 d of rewatering, but it remains to be seen whether additional drought events might lead to maintained high GB concentrations and thus lower sugar yields.

### Metabolic adjustment occurred at the expense of regrowth

Drought affects plant growth and yield and even short-term water deficits can induce significant yield losses in sugar beet, particularly when arising during early development [38]. Here, plants resumed growth under rewatering, but maintained a lower growth rate in both organs until the end of the experiment, suggesting a need for allocating C to metabolic adjustment, continued energy supply and an efficient damage repair after cessation of the stress.

The transient increase in root biomass compared to controls during the final days of drought was mainly due to the progressive formation of a network of fine side roots, rather than an increase of the taproot (data not shown). A dense mat of side roots increases the soil volume that can be exploited for water, but redirection of resources into side roots occurs at the expense of taproot formation and ultimately reduced incorporation of sucrose [39]. It is therefore likely that an early impairment of the taproot formation might contribute to sucrose yield losses at harvest [38], and it remains to be determined whether this can be compensated during development, especially if future drought spells arise.

### Drought-induced carbon re-allocation is only partly reversed during rewatering

Under drought, C allocation patterns are changed in order to distribute resources to the sites where they are most needed during acclimation and, after stress release, restauration processes [40]. Here, drought led to elevated levels of soluble sugars in leaves, which were paralleled by a decrease in starch. Decreasing starch levels in drought-stressed leaves have been previously observed in sugar beet [41] and spinach [42], and result from an inhibited starch biosynthesis [43] or enhanced turnover to provide soluble sugars for OA [44].

Rewatering reversed the drought induced changes of sucrose and starch, clearly indicating that photosynthesis quickly recovered after rewatering. Interestingly, starch levels reached and maintained values which were significantly higher than the controls throughout the second half of the rewatering period. Since growth resumed only after d 18 (5 DAR) and at reduced rates, it is possible that inhibited growth contributed to the observed accumulation of starch. It would be interesting to see whether starch concentrations return to control levels after a longer recovery period, or whether this is a long-lasting stress imprint affecting sugar metabolism throughout development. Alternatively, starch synthesis and degradation follow a circadian regulation, which can be compromised under drought [45]. Under regular water supply, leaves accumulate starch during the day and remobilize it at night to support metabolism and growth [42]. It cannot be ruled out that drought-induced perturbations of the diurnal pattern of starch metabolism were involved in the observed starch accumulation during rewatering, and further studies should include measurements of diurnal starch variations.

### Amino acids accumulate during drought and respond differently to rewatering in leaves and roots

The PCA indicates that AA represented the dominant loadings under severe stress in both roots and leaves (Fig 4). In other words, the increase in AA was indicative for the transition from mild to severe drought stress. Increasing AA concentrations, and especially AAAs and BCAAs, were frequently observed under drought in leaves and roots of several species [46–49]. Due to their slow catabolism, these AA represent an excellent pool to rebuild proteins after the stress ends. The AA pool can be fed either by N assimilation, or by chlorophyll and protein turnover. Here, the increase in AA was accompanied by decreasing total protein concentrations in both organs, which might indicate enhanced proteolysis provoked by the stress in combination with a slow catabolism of AA [50]. However, the AA accumulation under drought was preceded by a significant drop in nitrate concentrations (Fig 3, S5 Fig). Since nitrate-supply and uptake into the root are likely inhibited under drought [51], such a drop in plant nitrate levels could be an indicator for continued N-assimilation, at least during the first days of drought and as long as the nitrate-pool was not exhausted. Indeed, this would be an excellent valve to get rid of excess energy caused by drought-induced growth inhibition, while photosynthesis is still functioning. However, other studies indicate rapid inhibition of NR-activity under drought in different species [52,53]. Additional experiments are under way to assess N-assimilation and NR-activity in drought stressed sugar beet.

Under rewatering, levels of BCAAs and AAAs returned to control levels within several days, but more slowly in roots compared to leaves. Under conditions of limited resources, BCAAs as well as AAAs play a role in mitochondrial respiration [54], and can be catabolized into the TCA cycle to contribute to the cellular energy metabolism [55]. In addition, BCAA-derived metabolites such as fatty acids and acyl sugars contribute to plant growth, defense and flavor [56], which may be beneficial for the recovery process. The rather rapid decrease of BCAA, aspartate, asparagine and phenylalanine levels in leaves during rewatering might indicate that these AAs were important in contributing to the energy supply in photosynthetic tissues, which were severely damaged by ROS formation, while newly assimilated C was first used to repair essential structures before being translocated to roots. Alternatively, longer maintenance of elevated AA levels in roots might be attributed to an overall slower recovery of protein synthesis and growth in belowground organs.

Surprisingly, AAAs only transiently returned to control levels in leaves, and a second strong increase was evident towards the end of the rewatering period, which was not observed in roots. Aromatic AAs serve as precursors of secondary metabolites including anthocyanins, which in turn are precursors of lignin and suberin, and auxin, which plays a leading role in plant organ formation. At this point it can only be speculated that the second increase in AAAs in leaves could indicate an increased demand for these substances during the onset of regrowth or represents a stress imprint, which might confer a competitive advantage during future drought events [7].

Another difference between roots and leaves was the stronger drought-induced increase of serine in roots. Serine is involved in various biological processes such as cell proliferation, C-1 metabolism, signaling and sphingolipid biosynthesis and serves as precursor for tryptophan biosynthesis [57]. Hence, sufficient serine concentrations are fundamental for all tissues to ensure plant development, and evidence for its involvement in abiotic and biotic stress responses is increasing [55]. However, an explanation for the observed additional increase towards the end of the rewatering period is currently not known.

## Conclusions

The untargeted ^1^H-NMR metabolomic approach delivered a detailed metabolic picture of temporarily drought stressed *Beta vulgaris* plants. Drought-induced changes in primary metabolism as well as impairments of plant water status and membrane stability were mostly reversed within 12 d of recovery, but clearly different recovery dynamics were observed in roots and leaves, possibly related to the distinct functions and the need for efficient recovery strategies in each organ. This difference is reflected in the PCA results, which indicated that roots sampled at the end of the rewatering period were metabolically distinct from non-stressed plants, while this was not the case in leaves. Only in leaves we detected a second increase in AAAs towards the end of rewatering. At this point it remains unclear whether this indicates an increased demand for AAAs during the onset of regrowth, or whether it represents a stress imprint which might be beneficial during an upcoming drought spell.

Damage repair seemed to be particularly important during the initial recovery phase. The late increase of GB and proline towards the end of the drought period especially in leaves might indicate their protective function specifically for the maintenance of favorable conditions for cellular restauration.

Even though the targeted analysis of further metabolites such as NO_3_^-^ indicated a continued N-assimilation at least during the initial days of drought, metabolic adjustments and repair processes during recovery occurred at the expense of growth for at least 12 d. Whether this reduced growth rate or perturbation in the diurnal starch metabolism accounted for the observed significant increase in starch during the recovery period still awaits verification.

Overall, it can be concluded that drought and recovery are two distinct processes subject to different regulatory mechanisms actively driven by the plant. While progressive drought leads to acclimation processes required for a new metabolic steady-state under increasing water limiting conditions, rewatering results in a re-distribution of resources to ensure the recovery process, in an organ specific manner.

## Supporting information

S1 Fig Harvest scheme of the young *Beta vulgaris* plant. Overview of the entire plant (A) and how leaves were sampled and prepared for further processing (B).
(TIF)

S2 Fig RGB images of well-watered, stressed and rewatered young *Beta vulgaris* plants. Pictures were taken at days 13, 15 and 25 (A, C, E; well-watered plants), with the respective drought-stressed (B) and rewatered plants (D, F).
(TIF)

S3 Fig Two representative ^1^H-NMR spectra. The spectra show different magnifications of polar extracts of leaves (A) and roots (B) of well-watered *Beta vulgaris* plants at day 15 of the experimental period. Numbers in the left upper corner correspond to the magnification of the selected section. Resonances are annotated according to Tables 1 and 2.
(TIF)

S4 Fig Changes of sucrose and starch under well-watered conditions and temporary drought. Sucrose (A) and starch (B) under well-watered conditions (closed circles) and temporary drought (open circles). C: Difference to the control of starch (filled triangles) and sucrose (open squares) concentrations. All values are means ± s.e. (n = 4). Asterisks indicate significant differences to the control plants (Duncan, α = 0.5, P < 0.05).
(TIF)

S5 Fig Nitrate (A, B), total amino acids (C, D) and total protein (E, F) concentrations of sugar beet leaves (A, C, E) and roots (B, D, F) under well-watered conditions and temporary drought. Closed circles indicate well-watered conditions and open circles indicate temporary drought (open circles). All values are means ± s.e. (n = 4). Asterisks indicate significant differences to the control plants (Duncan, α = 0.5, P < 0.05).
(TIF)
