# Supplementary figures and images for: 1H-NMR metabolomic profiling reveals a distinct metabolic recovery response in shoots and roots of temporarily drought-stressed sugar beets
Source: PLoS One. 2018 May 8;13(5):e0196102. doi: 10.1371/journal.pone.0196102 (PMC5940195; doi:10.1371/journal.pone.0196102)

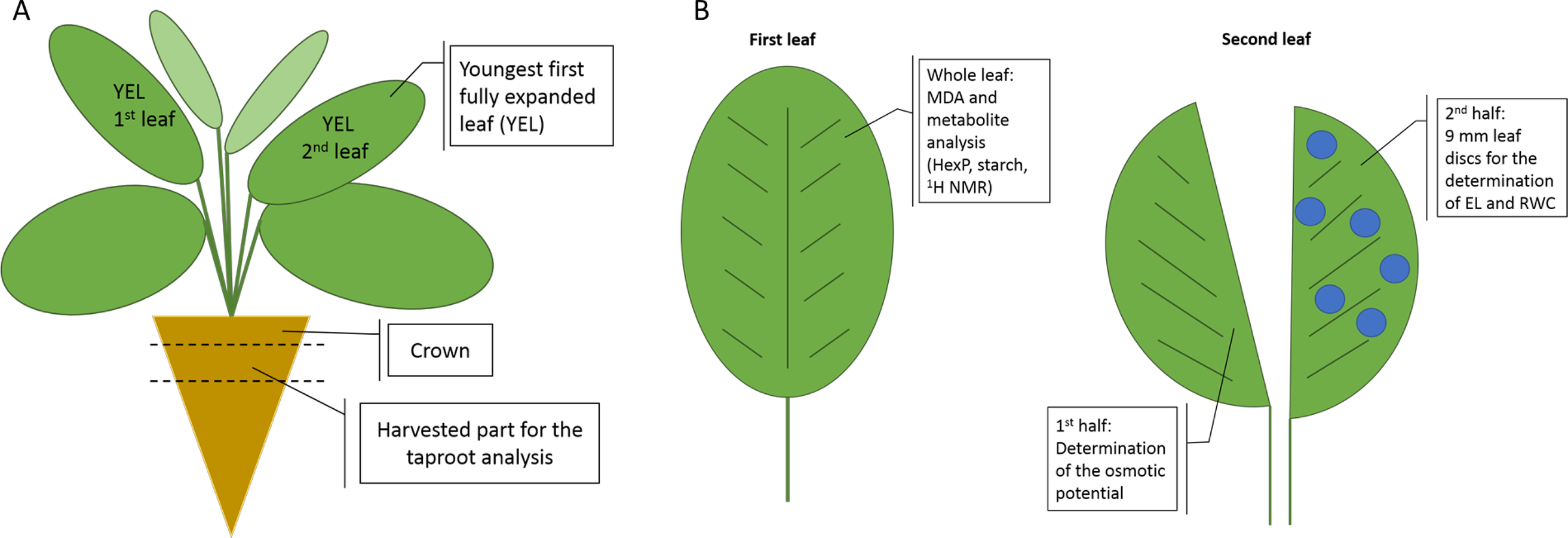

Supplement: S1 Fig — Overview of the entire plant (A) and how leaves were sampled and prepared for further processing (B). (TIF) [file pone.0196102.s001.tif]

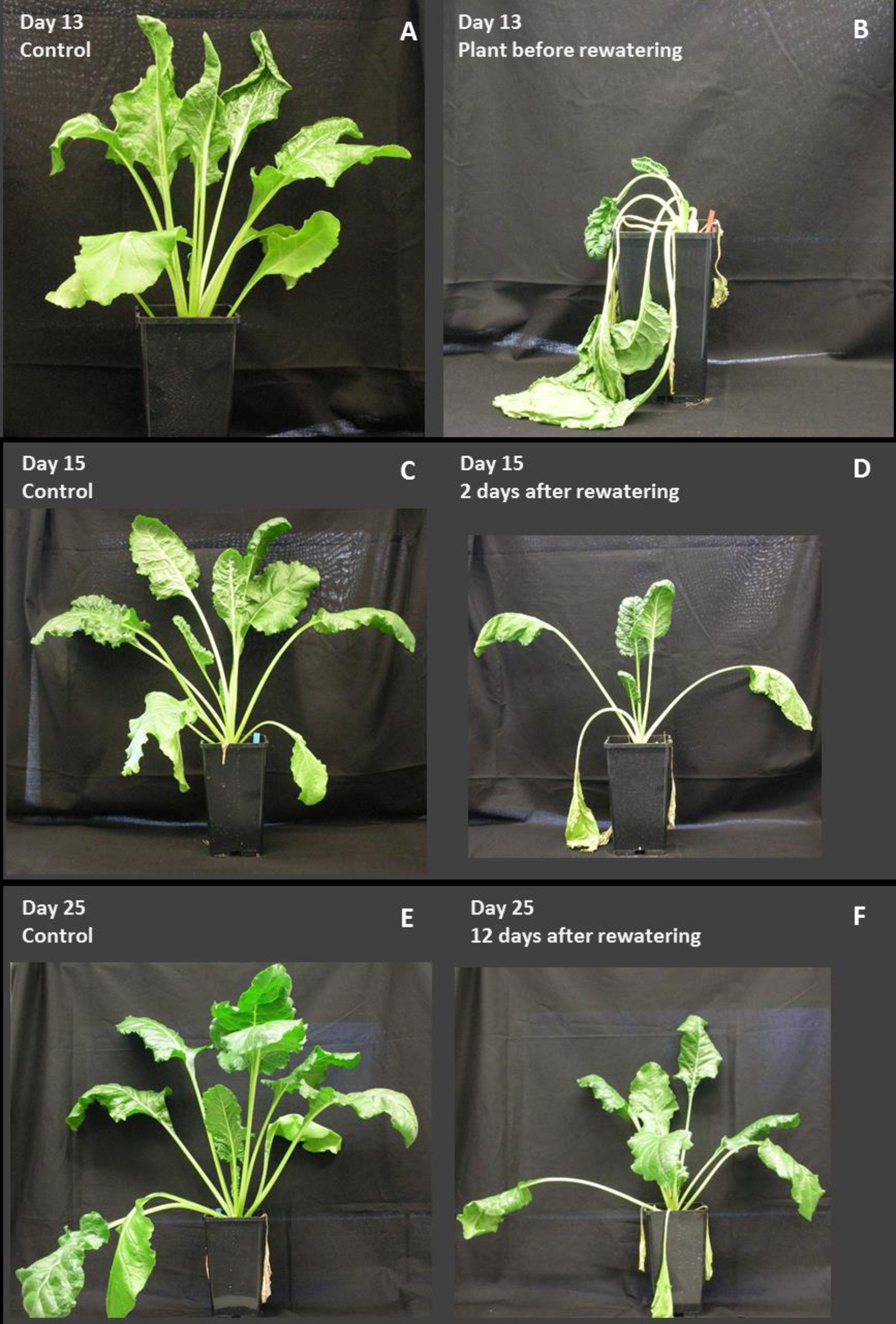

Supplement: S2 Fig — Pictures were taken at days 13, 15 and 25 (A, C, E; well-watered plants), with the respective drought-stressed (B) and rewatered plants (D, F). (TIF) [file pone.0196102.s002.tif]

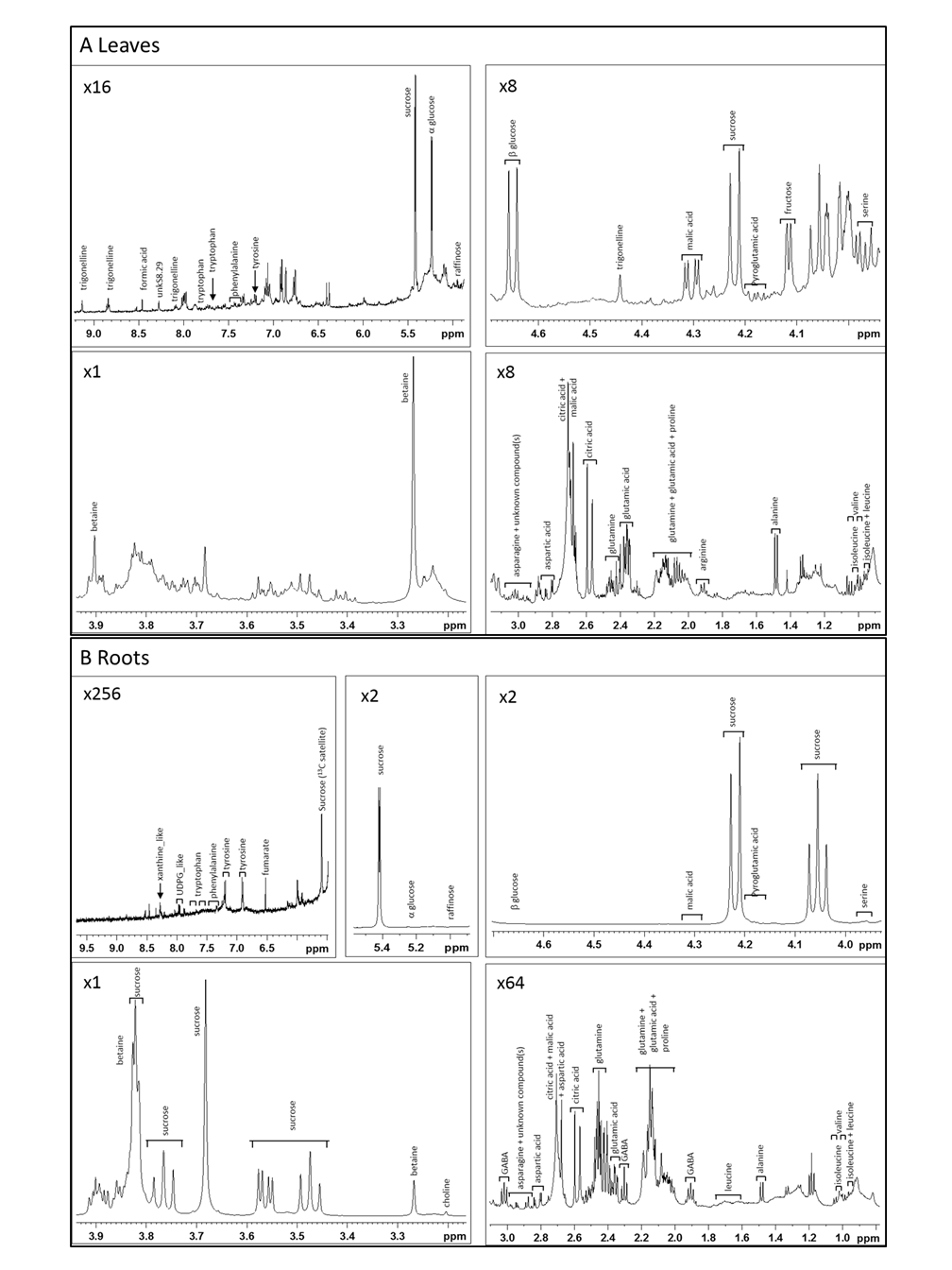

Supplement: S3 Fig — The spectra show different magnifications of polar extracts of leaves (A) and roots (B) of well-watered Beta vulgaris plants at day 15 of the experimental period. Numbers in the left upper corner correspond to the magnification of the selected section. Resonances are annotated according to Tables 1 and 2. (TIF) [file pone.0196102.s003.tif]

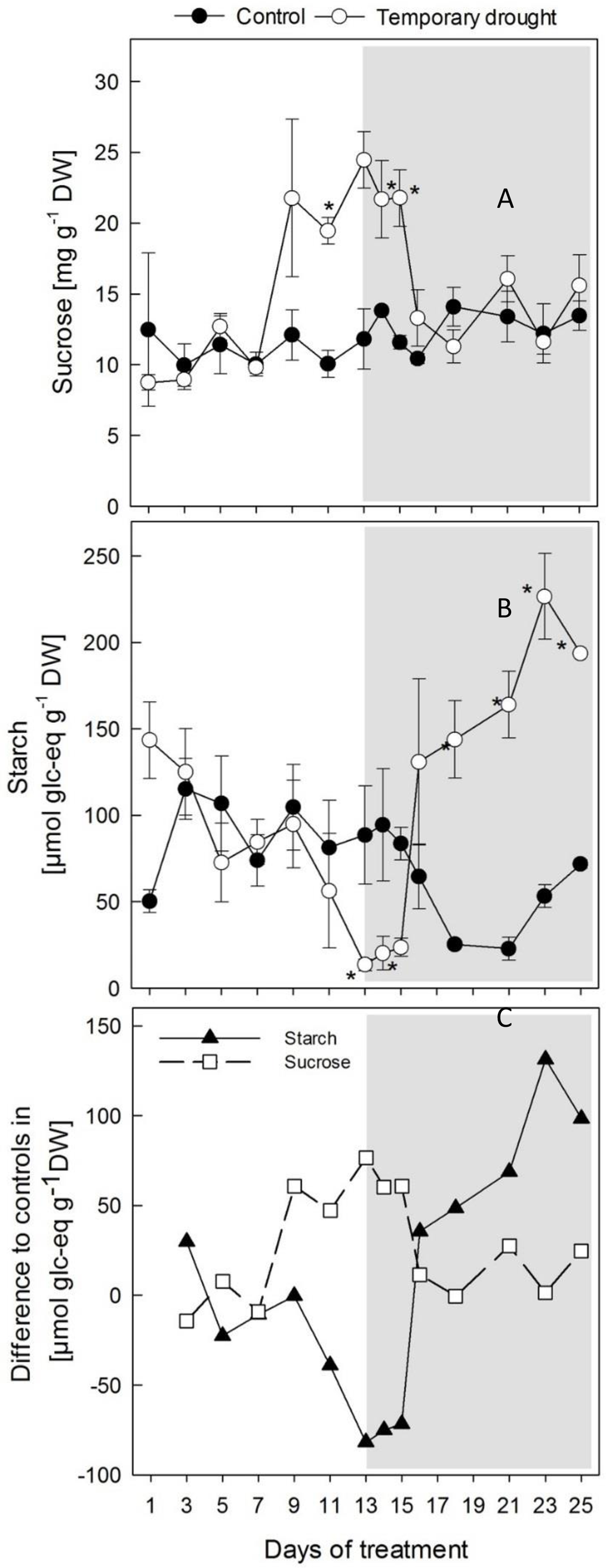

Supplement: S4 Fig — Sucrose (A) and starch (B) under well-watered conditions (closed circles) and temporary drought (open circles). C: Difference to the control of starch (filled triangles) and sucrose (open squares) concentrations. All values are means ± s.e. (n = 4). Asterisks indicate significant differences to the control plants (Duncan, α = 0.5, P < 0.05). (TIF) [file pone.0196102.s004.tif]

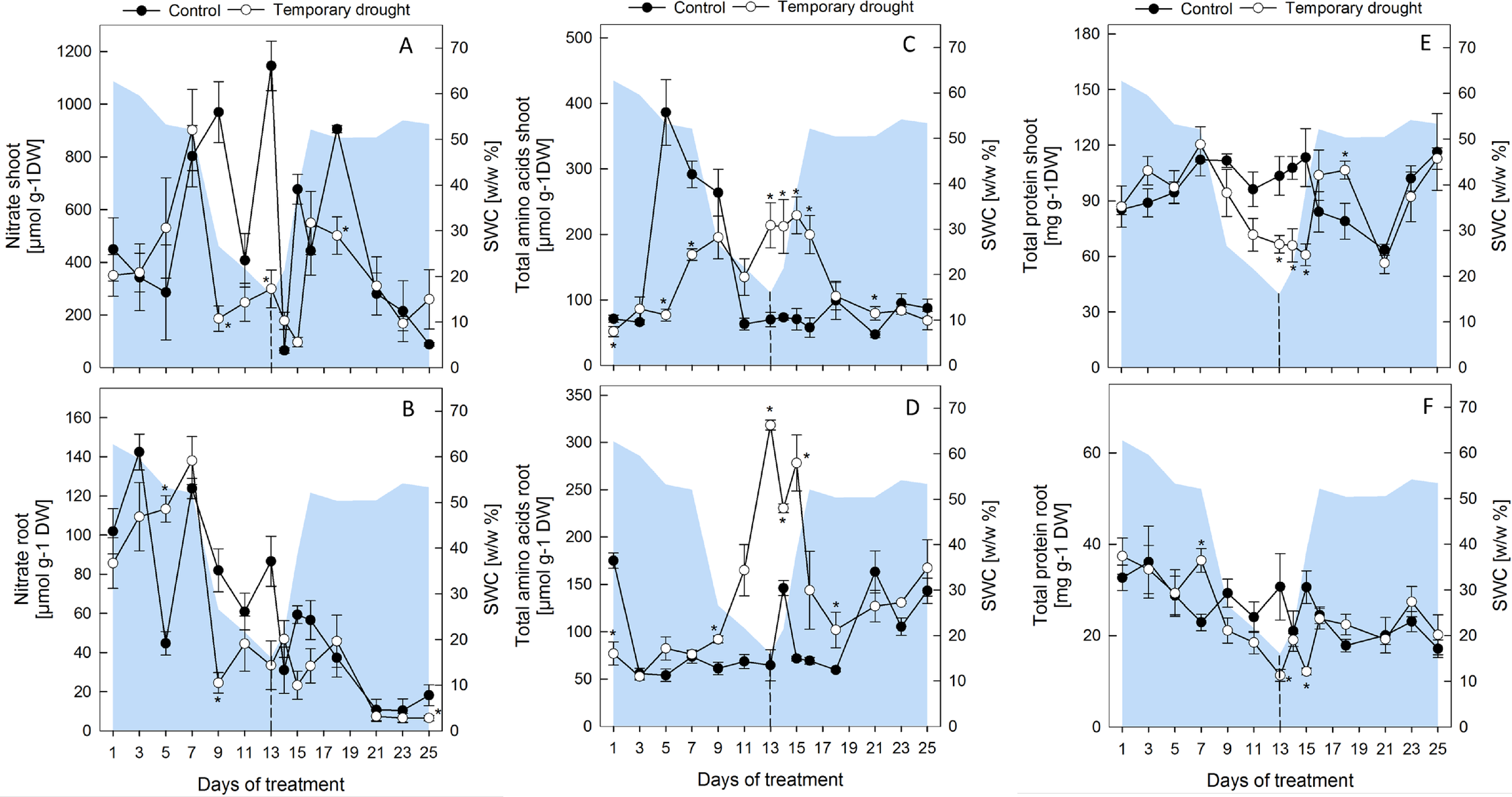

Supplement: S5 Fig — Closed circles indicate well-watered conditions and open circles indicate temporary drought (open circles). All values are means ± s.e. (n = 4). Asterisks indicate significant differences to the control plants (Duncan, α = 0.5, P < 0.05). (TIF) [file pone.0196102.s005.tif]
